# Factors affecting dural sinus density in non-contrast computed tomography of brain

**DOI:** 10.1038/s41598-019-48545-y

**Published:** 2019-08-19

**Authors:** Reza Akhavan, Bita Abbasi, Moein Kheirollahi, Afshar Ghamari Khameneh, Jahanbakhsh Hashemi, Samin Khoei, Gisoo Darban Hosseini Amirkhiz

**Affiliations:** 1 Department of Emergency Medicine, Faculty of Medicine, Mashhad University of Medical Sciences, Mashhad, Iran; 2 Department of Radiology, Faculty of Medicine, Mashhad University of Medical Sciences, Mashhad, Iran; 3 Tehran University of Medical Sciences, Tehran, Iran

**Keywords:** Brain imaging, Neurology

## Abstract

The possibility of changing the intracranial vasculature computed tomography (CT) attenuation under the influence of variable factors is a long-held unestablished belief. The purpose of this study is to evaluate factors affecting dural sinus density in non-contrast computed tomography of the brain. Patients presented with acute neurologic symptoms to the emergency department were candidates to be enrolled in this study. A region of interest (ROI) measuring 1–2-mm^2^ recorder (base on sinus size) used to measure the attenuation of each sinus in Hounsfield Unit (HU) and then mean density calculated. CBC, BUN and Cr were extracted from patients’ records. Chi-square test, correlation analyze, independent sample unpaired student t-test and one-way ANOVA test and Multivariate logistic regression were used. Positive significant correlation (0.48) was found between the hematocrit level (HCT) and average attenuation in the four sinus segments (P value < 0.0001) and between the HCT and basilar artery attenuation (P value < 0.0001). There was no significant correlation between the age and average attenuation. There was a significant and negative correlation between the BUN/Cr and average attenuation. Using a multivariate analysis on a large sample volume, we conclude that Hgb and HCT are the only factors that have a significant correlation with average sinus attenuation. This correlation is relatively stronger for Hgb in comparison to HCT.

## Introduction

Cerebral venous sinus thrombosis (CVST) is an uncommon but important cerebrovascular disease which accounts for 0.5–1% of cases of all strokes and is associated with up to 10% mortality^1,2^. The disease predominantly affects young adults^3^ and may lead to adverse outcomes including infarct and brain hemorrhage. Although the prognosis is highly dependent on timely diagnosis and prompt anticoagulation therapy^4,5^, the insidious onset and non-specific complaints cause an average 7-day delay from the symptoms to the diagnosis^6–9^. Non-contrast computed tomography (NCCT) is widely used as the initial imaging modality of choice in neurological emergencies^2,10–13^. Increased attenuation of cerebral sinus venous structures is considered the only direct finding of acute CVST in the NCCT^11,14,15^. Increased sinus attenuation has the advantage of being objective and easily calculated, while being able to diagnose CVST in the acute stage, when treatment is most likely to be effective and associated with favorable outcome^13^. The drawback of this sign is the influence of various factors like serum hematocrit level and dehydration on sinus attenuation that may lead to false positive results and inappropriate disposition^2,10,11,15^. The possibility of changing the intracranial vasculature computed tomography (CT) attenuation under the influence of variable factors is a long-held unestablished belief. A better understanding of these factors can lead to better triage of the patients and proper treatment of the disease. The purpose of this study is to evaluate factors affecting dural sinus density in non-contrast computed tomography of the brain.

## Methods

This retrospective study was approved by the Chancellor of Research, Mashhad University of Medical Sciences (approval ID: IR.MUMS.fm.REC.1395.176). The informed consent was waived by the Ethics Committee, as the study was retrograde. This study was carried out according to relevant guidelines and regulations.

### Patient selection

Patients presented with acute neurologic symptoms to the emergency department of our uni- versity hospital in the time period between June 2018 to March 2019 were candidates to be en- rolled in this study. Clinical assessment, laboratory investigations, and NCCT of the brain were reviewed for each patient. Inclusion criteria included newly onset neurological symptoms, NCCT performed at presentation, complete blood count (CBC), blood urea nitrogen (BUN) and serum creatinine (Cr) evaluation within 24-hours of NCCT. In all patients, diagnosis of CVST was excluded by magnetic resonance venography (MRV), or established diagnosis other than CVST. Exclusion criteria were the presence of artifacts in NCCT, any intracranial pathology that prevented proper measurement of venous sinus attenuation (like intracranial hemorrhage, skull fracture in the vicinity of dural sinuses, increased intracranial pressure, intra or extra axial mass and recent brain surgery), intravenous contrast administration or blood transfusion within the previous 48 hours, and age younger than 6 months (to eliminate the effect of the fetal hemoglobin). A total of 511 patients were finally included in the study.

### Data interpretation

**Figure 1:**
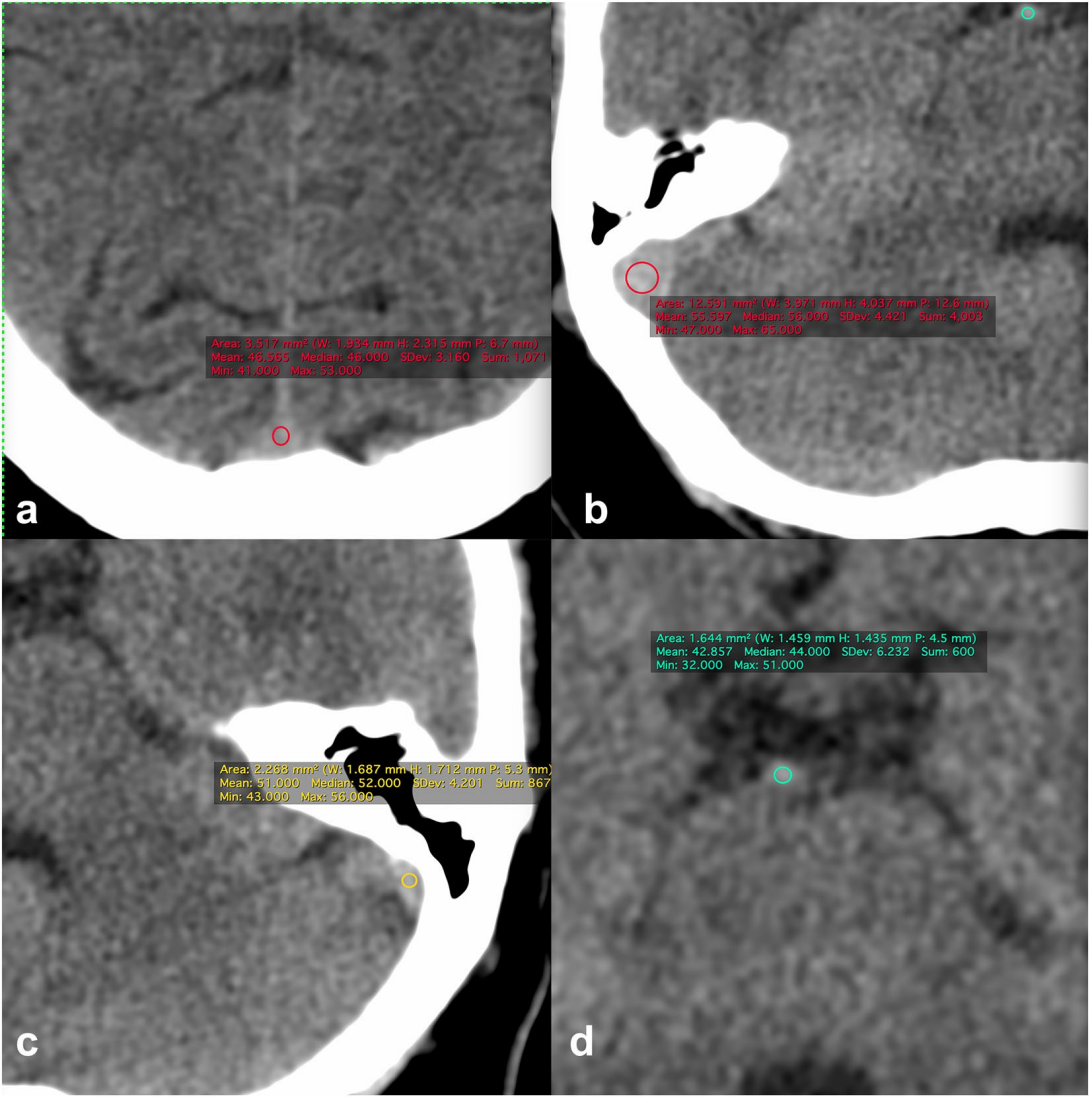
Zoomed in images of axial brain CT scans shows blood density measurement in the superior sagittal (**a**), right sigmoid (**b**), left sigmoid (**c**) sinuses and basilar artery (**d**). All images are first zoomed in, and a ROI is placed in the center of vascular structure and is not in contact with the vessel wall.

Attenuation of venous sinuses was measured by one specialized radiologist to reduce inter- observer variability. A region of interest (ROI) measuring 1-2-mm^2^ (based on sinus size) was used to measure the attenuation of each sinus in Hounsfield Unit (HU) and then mean density was calculated. 1984 venous sinuses including 514 superior sagittal venous sinuses, 513 Torcula Herophilis,484 right sigmoid venous sinuses, 473 left sigmoid venous sinuses and 488 basilar arteries were evaluated (Fig. 1). Mean HU in the basilar density was measured as an additional measurement for blood density. Some studies have tried to normalize the mean density in the dural veins according to the hematocrit level or the mean density in other intracranial vessels to increase false positive results of CVST diagnosis. As the basilar density has lower blood flow rates compared to the other arteries in the Willis circle and is therefore more physiologically similar to dural veins, we decided to measure the mean density in this vascular structure as an additional measurement. CBC, BUN and Cr were extracted from patients’ records. The Hounsfield number of superior sagittal sinus to HCT level (H:H ratio, Hounsfield unit-to- hematocrit ratio) was calculated.

### Image acquisition

All images were done via Neusoft^TM^ 16-slice CT scanner with the following parameters:180–450 mAs, 120 kV and section thickness of 5 mm.

### Statistical analysis

Using the IBM SPSS Statistics for Windows, Version 25.0. Armonk, NY: IBM Corp, we analyzed our data. After reviewing the normal distribution of quantitative data, Chi-square test, correlation analyzes, independent sample unpaired student t-test and one-way ANOVA test and Multivariate logistic regression were used. P value < 0.05 considered statistically significant.

## Results

**Figure 2:**
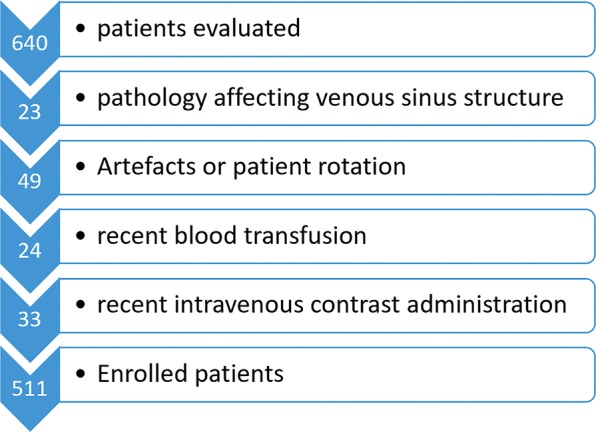
Flow chart showing the stages of patient selection.

A total of 640 patients were evaluated in this study. Twenty-three patients were excluded due to the presence of a pathology affecting the venous sinus attenuation measurement (hematoma or infarction in the vicinity of venous sinuses, severe cerebral edema or masses adjacent to the venous sinuses). In 49 patients, the presence of severe artifacts in the vicinity of the dural sinuses prevented the measurement of attenuation or CT scan sections were not properly prepared (rotation of the patient or section other than orbitomeatal). Twenty-four patients were excluded due to recent blood transfusion and 33 patients were excluded due to recent IV contrast media administration. Finally, 511 patients were enrolled in the study (Fig. 2). The mean age was 61.58 (ranged from 4 to 99 years) without significant gender difference (mean age for men was 62.03 years and for women was 60.99 years).

### HCT/Hgb

**Figure 3:**
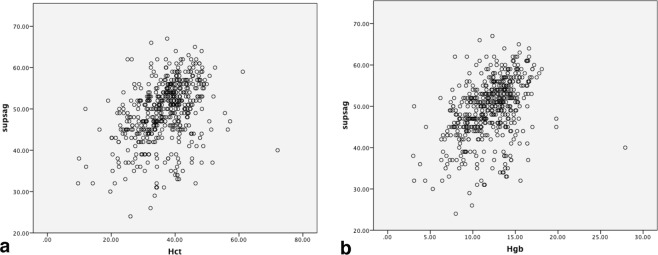
Graph illustrating correlation between average attenuation in the superior sagittal sinus and HCT and Hgb (**b**). supsag: average attenuation in the superior sagittal sinus. HCT: hematocrit level. Hgb: Hemoglobin concentration.

The mean hematocrit level (HCT) for our sample population was 36.76 (ranged from 9.6 to 72.1) whereas for hemoglobin (Hgb) the mean was 12.03 (ranged from 3 to 27.9) and for RBC count the mean was 4.17 (ranged from 1.15 to 9.3). No significant correlation was found between age and HCT (P value: 0.808); there was still no significant correlation upon controlling Cr (P value 0.07). A positive significant correlation (0.48) was found between the HCT and average attenuation in the four sinus segments (P value < 0.0001) and between the HCT and basilar artery attenuation (P value < 0.0001). Average attenuation had a little stronger correlation with Hgb than HCT (Table 1, Fig. 3).

**Table 1:** The correlation between average attenuation in the vascular structures with HCT and Hgb.

Vascular structures	Correlation with HCT		Correlation with Hgb	
	P value*	R	P value*	R
Superior sagittal sinus	0.000	0.380	0.000	0.416
Torcula Herophili	0.000	0.449	0.000	0.484
Right sigmoid sinus	0.000	0.498	0.000	0.528
Left sigmoid sinus	0.000	0.519	0.000	0.555
Basilar artery	0.000	0.410	0.000	0.411

*Pearson’s test.

The patients were then divided according to their blood parameters to the anemic, normal and polycythemia groups (Table 2). The normal Hgb range defined as 13.5 to 17.5 grams(g) per deciliter(dl) of blood for men and 12 to 15.5 g/dl for women. The normal HCT level considered between 34.9 and 44.5 percent for adult women and 38.8 to 50 for adult men. The normal values for children vary depending on the child’s age and sex. Lower and upper than normal levels indicate anemia and polycythemia, respectively.

**Table 2:** Description of blood parameters.

Groups	^#^patients (%)	HCT (SD)	Hgb (SD)	RBC (SD)
Anemic	284	31.55 (5.74)	10.18 (2.099)	3.61 (0.75)
Normal	200	42.45 (3.32)	14.13 (1.32)	4.79 (0.46)
Polycythemia	26	49.90 (6.2)	16.11 (2.98)	5.51 (1.00)

SD = Standard deviation, HCT = hematocrit, Hgb = hemoglobin, RBC = red blood cells.

There was a significant difference in the mean attenuation of all four dural vein segments and basilar artery in these three groups (p values < 0.001, One-way ANOVA test). However, there was considerable overlap in the average attenuation values of dural veins in these three groups, we could not find an optimum HU cut-off for detecting anemia and polycythemia in our sample volume (Fig. 3).

To calculate the H:H ratio, we used the attenuation values of superior sagittal sinus. The mean H:H ratio was 1.4 (ranged from 0.55 to 4.2).

### Age

The mean age was 61.58 years (20.26 SD). There was no significant correlation between the age and average attenuation in superior sagittal, Torcula Herophili, right and left sagittal sinuses and basilar artery with P value of 0.331, 0.101, 0.064, 0.073 and 0.792 respectively.

### BUN/Cr ratio

The mean BUN/Cr ratio was 18.07 (ranged from 1.5 to 110). There was a significant and negative correlation between the BUN/Cr and average attenuation in superior sagittal, Torcula Herophili, right and left sagittal sinuses and basilar artery with p values of 0.004, 0.000, 0.002, 0.008 and 0.01 respectively. We also found a significant negative correlation between BUN/Cr and HCT level (P value: 0.001). The significant correlation between BUN/Cr and average attenuation in the four sinus segments disappeared upon controlling for HCT level.

On a multivariate test, Hgb level correlated significantly with average attenuation in all four sinus segments and basilar artery (P value < 0.001). HCT level showed a significant correlation with the average attenuation in superior sagittal and Torcula Herophili (p values 0.041 and 0.046 respectively), but not with other vascular structures. No significant correlation was seen between the BUN, Cr and BUN/Cr and average attenuation in any of the vascular structures.

## Discussion

NCCT is a cost-effective and widely available modality that is used as the initial imaging of choice in many of the now-onset neurological symptoms^11^. The only direct sign of acute CVST in NCCT is increased attenuation within the dural veins, which has a reported sensitivity of 73%^6,16^. Although, a prompt diagnosis of CVST is critical for starting appropriate treatment and preventing sequelae^4,13^, false positive diagnosis can on the other hand mask the correct diagnosis and lead to patient’s anxiety and inappropriate treatment strategies. Polycythemia is proposed in many studies as a possible source of false positive NCCT interpretations^17^. In this study we used a large sample volume of patients to assess the factors affecting dural vein attenuation, trying to propose the best method for normalizing absolute average HU to reach the maximum possible sensitivity for NCCT in the diagnosis of acute CVST.

In the multivariate study, HCT and Hgb were the only factors that had a significant corre- lation with average sinus attenuation, and Hgb had a slightly stronger correlation. A significant association between sinus attenuation and HCT and Hgb level has long been established^18^. Taking this correlation into account, Black *et al*.^14^ suggested H:H ratio as a normalizing parameter for objective evaluation of CVST in the NCCT and recommended an H:H ratio greater than or equal to 2 as a cutoff value to separate patients with CVST from those without. A subsequent study by Buyck *et al*.^11^ suggested H:H ratio greater than 1.52 as a cutoff value for acute CVST. In our study, the mean H:H ratio was 1.40, ranging from 0.55 to 4.2. In our patients, 134 (26.1%) had H:H ratio greater than 1.52, and 23 (4.5%) had H:H ratio equal or greater than 2. This shows that both of these suggestions might be accompanied by unacceptable rates of false positive results. Our study showed that average HU had a stronger correlation with Hgb than the HCT. This finding is also appreciated in research by AlRyalat *et al*.^10^. This suggests that redefining H:H ratio as Hounsfield-Hgb ratio might be helpful in increasing the sensitivity of NCCT readings.

Black *et al*.^14^ suggested that average HU of greater than 70 is suspicious for acute CVST and warrants further evaluation. Buyck *et al*.^11^ suggested the HU of greater than 62 to be further evaluated. In our sample, 10 patients (2%) had average HU greater than 62, 5 (1%) had average HU greater than 65, and only 3 (0.6%) had average HU greater than 70 in the superior sagittal sinus. This suggests that using absolute sinus HU as a criterion for the diagnosis of acute CVST is associated with a smaller number of false positive results in comparison to H:H ratio. This finding is also supported by Alryalat *et al*.^10^. We also suggest further evaluation in patients with average sinus attenuation greater than 65 HU.

**Figure 4:**
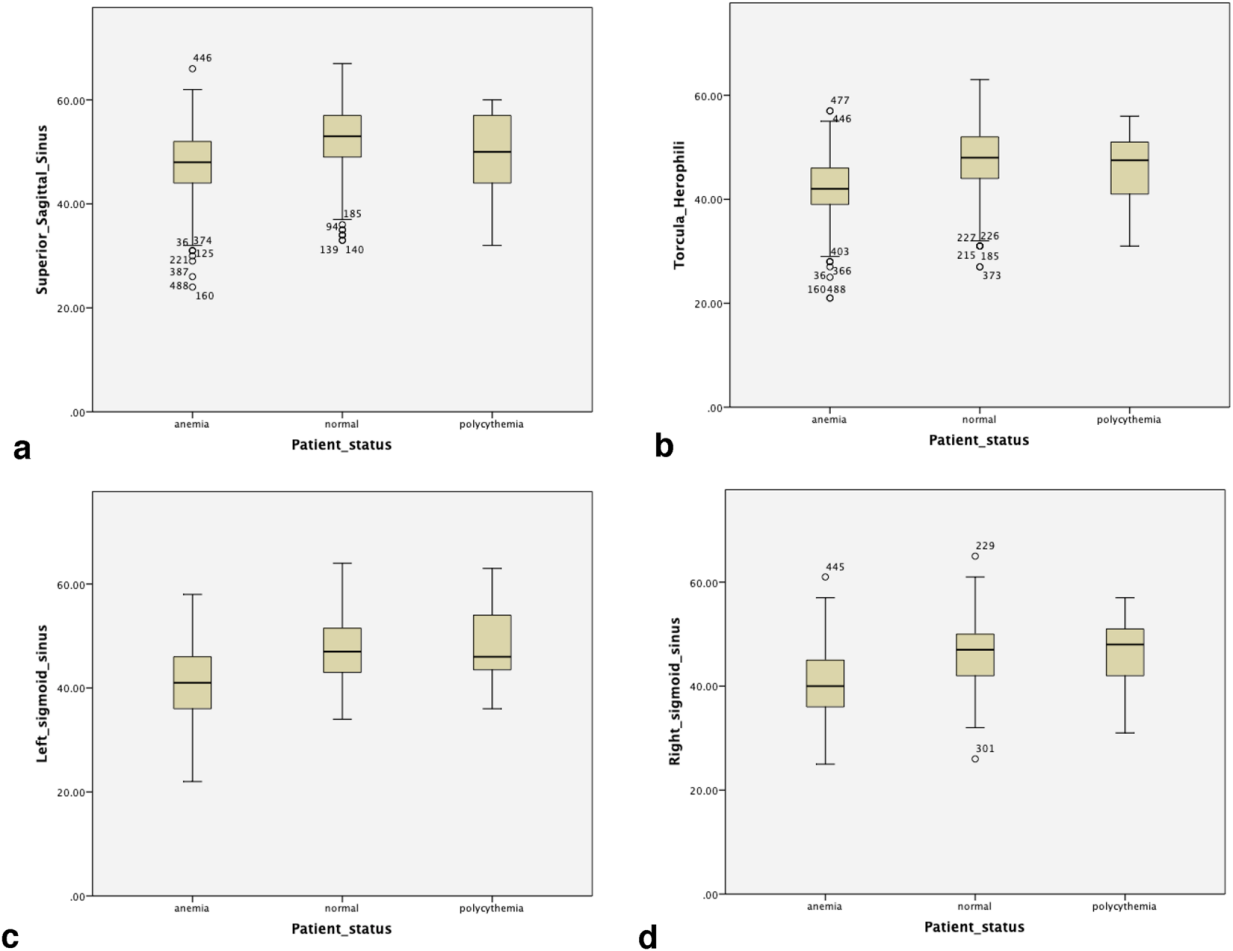
Graphs comparing the average HU in the anemic, normal and polycythemic patients. (**a**) superior sagittal sinus, (**b**) Torcula Herophili, (**c**) left sigmoid sinus, (**d**) right sigmoid sinus. There is considerable overlap of the average attenuation between the three groups.

Our study included both anemic and polycythemic patients in addition to patients with nor- mal Hgb concentration. As there was considerable overlap in the average attenuation values of dural veins in these three groups, we could not find an optimum HU cut-off for detecting anemia and polycythemia in our sample volume (Fig. 4).

After removing the effect of HGB and HCT on attenuation, there was no statistically sig- nificant correlation between age and average sinus attenuation. Lee *et al*.^19^ and Al-Rayat *et al*.^10^ both found a weak correlation between the age and average HU. We could not establish such a correlation.

We also did not find any significant difference in the average HU of vascular structures between male and female patients. This result contradicts the findings of AL Ryalat *et al*.^10^ and needs further evaluation.

In many studies, BUN/Cr is considered as a marker of dehydration and BUN/Cr greater than 20 is usually considered as dehydration^11,14,20^. Although a negative correlation was preliminarily found between the BUN/Cr and average HU, this correlation disappeared upon controlling the results for HCT. No significant correlation was found between the average HU in the vascular structures and neither BUN/Cr nor BUN or Cr level. We found a significant negative correlation between BUN/Cr and HCT. This finding may be justified by the presence of comorbidities, and the greater likelihood of dehydration in the anemic patients. This should also be kept in mind that dehydration is not the only cause of increased BUN/Cr ratio, and some other conditions like parenteral nutrition and steroid therapy can elevate the BUN/Cr ratio.

There are some limitations in our study. We used BUN/Cr ratio as the marker of dehydra- tion in our patients, while the best marker is clinical status. This limitation happened due to the retrospective design of our study and a lack of proper data in the subjects’ files. In spite of this limitation, our study represents an insight about the main confounding factors of one of the most important signs of CVST (Hyperattenuation).

## Conclusion

Using a multivariate analysis on a large sample volume, we conclude that Hgb and HCT are the only factors that have a significant correlation with average sinus attenuation. This correlation is relatively stronger for Hgb in comparison to HCT. Due to the vital role of NCCT as a screening modality in the new-onset neurologic symptoms, adding a quantitative measurement to the routine subjective evaluation is of paramount importance. We suggest that every patient with average sinus attenuation equal to or greater than 65 should be further evaluated for acute CVST. We believe that measuring sinus attenuation alone is sufficient and calculating H:H ratio would not increase diagnostic accuracy. This conclusion needs further confirmation with prospective studies.

## Data Availability

The datasets generated and analysis during the current study are available from the corresponding author on reasonable request.

## References

[1] Bousser MG, Ferro JM (2007). Cerebral venous thrombosis: an update. The Lancet. Neurology.

[2] Steven A, Raghavan P, Altmeyer W, Gandhi D (2016). Venous Thrombosis: Causes and Imaging Appearance. Hematol Oncol Clin North Am.

[3] Canhao P (2005). Causes and predictors of death in cerebral venous thrombosis. Stroke.

[4] Coutinho, J., de Bruijn, S. F., Deveber, G. & Stam, J. Anticoagulation for cerebral venous sinus thrombosis. *The Cochrane database of systematic reviews*, Cd002005, 10.1002/14651858.CD002005.pub2 (2011). 10.1002/14651858.CD002005.pub2 PMC7065450 21833941

[5] Hartel M (2015). Cerebral venous sinus thrombosis. Phlebology.

[6] Linn J (2009). Noncontrast CT in deep cerebral venous thrombosis and sinus thrombosis: comparison of its diagnostic value for both entities. AJNR. American journal of neuroradiology.

[7] Stam J (2005). Thrombosis of the cerebral veins and sinuses. The New England journal of medicine.

[8] Ferro JM, Canhao P, Stam J, Bousser MG, Barinagarrementeria F (2004). Prognosis of cerebral vein and dural sinus thrombosis: results of the International Study on Cerebral Vein and Dural Sinus Thrombosis (ISCVT). Stroke.

[9] Masuhr F, Mehraein S, Einhaupl K (2004). Cerebral venous and sinus thrombosis. Journal of neurology.

[10] Al-Ryalat NT (2016). Factors Affecting Attenuation of Dural Sinuses on Noncontrasted Computed Tomography Scan. J Stroke Cerebrovasc Dis.

[11] Buyck PJ (2013). CT density measurement and H:H ratio are useful in diagnosing acute cerebral venous sinus thrombosis. AJNR. American journal of neuroradiology.

[12] Zaheer S (2016). Quantitative non-contrast measurements improve diagnosing dural venous sinus thrombosis. Neuroradiology.

[13] Agrawal K, Burger K, Rothrock JF (2016). Cerebral Sinus Thrombosis. Headache.

[14] Black DF, Rad AE, Gray LA, Campeau NG, Kallmes DF (2011). Cerebral venous sinus density on noncontrast CT correlates with hematocrit. AJNR. American journal of neuroradiology.

[15] Alsafi A, Lakhani A, Carlton Jones L, Lobotesis K (2015). Cerebral Venous Sinus Thrombosis, a Nonenhanced CT Diagnosis? Radiology research and practice.

[16] Roland T (2010). Unenhanced brain CT is useful to decide on further imaging in suspected venous sinus thrombosis. Clinical radiology.

[17] Healy JF, Nichols C (2002). Polycythemia mimicking venous sinus thrombosis. AJNR. American journal of neuroradiology.

[18] New PF, Aronow S (1976). Attenuation measurements of whole blood and blood fractions in computed tomography. Radiology.

[19] Lee SY, Cha SH, Lee SH, Shin DI (2013). Evaluation of the effect of hemoglobin or hematocrit level on dural sinus density using unenhanced computed tomography. Yonsei medical journal.

[20] Liu CH (2014). Dehydration is an independent predictor of discharge outcome and admission cost in acute ischaemic stroke. European journal of neurology.

